# The Gut–Heart Axis: A Comprehensive Review of Microbiota’s Role in Cardiovascular Health and Disease and Emerging Therapeutic Strategies

**DOI:** 10.1155/crp/9920016

**Published:** 2026-02-09

**Authors:** Maneeth Mylavarapu, Angad Tiwari, Harshaman Kaur, Roopeessh Vempati, Harendra Kumar, Lakshmi Sai Meghana Kodali, Kiyan Ghani Khan, Sriharsha Dadana, Israel Garcia, Fabio Enrique Parada Cabrera, Amninder Singh, Sai Lakhan Kyasa, Vikramjit S. Purewal

**Affiliations:** ^1^ Department of Cardiology, Endeavor Health Cardiovascular Institute, Glenview, Illinois, USA; ^2^ Division of Cardiology, Department of Medicine, University of Chicago Pritzker School of Medicine, Chicago, Illnois, USA, uchicago.edu; ^3^ Department of Internal Medicine, Maharani Laxmi Bai Medical College, Jhansi, Uttar Pradesh, India; ^4^ Department of Internal Medicine, Honor Health Mountain Vista, Midwestern University, Mesa, Arizona, USA, midwestern.edu; ^5^ Department of Internal Medicine, Trinity Health Oakland Hospital, Pontiac, Michigan, USA; ^6^ Department of Internal Medicine, Dow University of Health Sciences, Karachi, Pakistan, duhs.edu.pk; ^7^ Department of Public Health, University of Michigan-Flint, Flint, Michigan, USA, msu.edu; ^8^ Department of Internal Medicine, Baqai Medical University, Karachi, Pakistan, baqai.edu.pk; ^9^ Department of Internal Medicine, Cheyenne Regional Medical Center, Cheyenne, Wyoming, USA, cheyenneregional.org; ^10^ Department of Family Medicine, University of Southern California School of Medicine, Los Angeles, California, USA, usc.edu; ^11^ Department of Cardiology, Instituto Guatemalteco de Seguridad Social (IGSS), Guatemala City, Guatemala; ^12^ Department of Cardiology, The Wright Center for Graduate Medical Education, Scranton, Pennsylvania, USA, thewrightcenter.org; ^13^ Department of Cardiology, TIMI Study Group, Brigham and Women’s Hospital, Boston, Massachusetts, USA, brighamandwomens.org; ^14^ Department of Internal Medicine, Honor Health Mountain Vista, Midwestern University, Mesa, Arizona, USA, midwestern.edu

**Keywords:** dysbiosis, endotoxemia, fecal microbiota transplantation (FMT), gut–heart axis, gut microbiota, next-generation probiotics (NGPs), short-chain fatty acids (SCFAs), trimethylamine N-oxide (TMAO)

## Abstract

This review examines the bidirectional relationship between the gut microbiota and cardiovascular diseases (CVDs), aiming to understand how microbial dysbiosis contributes to CVDs, including atherosclerosis, hypertension, and heart failure. Recent research emphasizes the gut microbiota’s role in modulating immunity via SCFAs and tryptophan metabolites, maintaining intestinal barrier integrity, and producing metabolites such as SCFAs (acetate, propionate, butyrate) and pro‐atherogenic TMAO. Dietary patterns, particularly the Mediterranean versus Western diet, significantly influence gut microbiota composition and CVD risk. Polyphenols and exercise have shown positive effects on gut microbiota and cardiovascular outcomes. A significant interplay exists between gut microbiota and cardiovascular health. Dysbiosis and metabolites like TMAO and LPS are implicated in CVD, while SCFAs and a balanced microbiota offer protection. Future research should focus on precision medicine, next‐gen probiotics, optimized FMT, and multiomics approaches to identify personalized CVD therapies.

## 1. Introduction

The human body exists in a dynamic symbiotic relationship with trillions of microorganisms, a concept that has gained prominence following the completion of the Human Genome Project in 2001 [1]. Since then, extensive research has uncovered the profound influence of the gut microbiota on various physiological processes, including metabolism, immunity, and organ system function. The gastrointestinal (GI) tract harbors an estimated 100 trillion microorganisms, primarily comprising bacterial phyla such as *Firmicutes* and *Bacteroidetes*, with smaller contributions from *Actinobacteria*, *Proteobacteria*, and *Verrucomicrobia* [2, 3]. Advances in molecular biology and sequencing technologies have revolutionized our understanding of these microbial communities, facilitating a deeper exploration of their impact on human health.

The gut microbiota is now recognized as a key player in cardiovascular (CV) health, with its metabolic byproducts influencing multiple mechanisms that regulate vascular function, lipid metabolism, and systemic inflammation [4]. Cardiovascular diseases (CVDs) remain a leading cause of global morbidity and mortality, with well‐established risk factors including hypertension, dyslipidemia, obesity, diabetes mellitus, and smoking [5]. However, emerging evidence suggests that gut microbiota composition and function are integral to CV health and disease [1, 2, 6–8]. The interplay between gut microbiota and CVD is largely mediated by microbial metabolites such as Short‐chain fatty acids (SCFAs), trimethylamine N‐oxide (TMAO), bile acids, and lipopolysaccharides (LPS), which influence inflammation, endothelial function, blood pressure regulation, and atherogenesis [7–10].

Gut dysbiosis, a disruption of microbial balance, has been implicated in the pathogenesis of various CVDs, including atherosclerosis, hypertension, and heart failure (HF) [9]. The composition of gut microbiota is shaped by multiple factors, including diet, age, genetics, antibiotic use, and environmental exposures. Western dietary patterns, characterized by high saturated fat, processed carbohydrates, and low fiber intake, have been associated with dysbiosis, increased TMAO production, and systemic inflammation, contributing to elevated CV risk. In contrast, Mediterranean and plant‐based diets, rich in fiber and polyphenols, promote a diverse gut microbiota, enhance SCFA production, and confer CV benefits [10–14].

Given the growing recognition of the gut microbiota’s role in CV health, microbiota‐targeted interventions are being explored as potential therapeutic strategies. Probiotics, prebiotics, dietary modifications, and pharmacological approaches targeting microbial metabolites are being investigated for their potential to mitigate CVD risk [13, 14]. The field of microbiome research is rapidly evolving, and future studies focusing on personalized microbiome‐based therapies hold promise for advancing CVD prevention and management [15–17].

This review aims to provide a comprehensive overview of the gut microbiota’s influence on CV health. By elucidating key microbial metabolites, underlying pathophysiological mechanisms, and potential therapeutic strategies, we highlight the need for precision medicine approaches incorporating microbiome‐based interventions to improve CV outcomes.

## 2. Methods

We performed a comprehensive literature search using PubMed and Google Scholar for articles published between 2000 and 2024, using the keywords “gut microbiota,” “cardiovascular disease,” “dysbiosis,” “TMAO,” “SCFAs,” and “gut–heart axis.” Relevance was assessed based on the quality of study design and contribution to the mechanistic understanding of the gut–heart axis.

## 3. Gut Microbiota: Composition and Function

The human gut microbiota, a complex community of microorganisms, significantly influences the host’s immune system, metabolism, and overall health [18]. This diverse ecosystem comprises numerous microorganisms, including bacteria, viruses, parasites, fungi, phages, eukarya, and archaea [1, 19]. Bacteria constitute the majority of the 10–100 trillion microorganisms within the human microbiome. More than 99% of these bacteria belong to the phyla *Firmicutes*, *Bacteroidetes*, *Proteobacteria*, and *Actinobacteria*, with *Firmicutes* and *Bacteroidetes* being the most prevalent groups in a healthy individual’s gut microbiota [20].

### 3.1. Composition

The human GI microbiome consists of distinct microbial communities that vary along the GI tract.

• Esophagus: The distal esophageal microbiome exhibits low diversity, with *Streptococcus* species being the dominant group. Other genera, including *Prevotella, Actinomyces, Lactobacillus, and Staphylococcus,* have also been identified [21].
• Stomach: Though historically considered inhospitable to bacteria, the stomach harbors *Helicobacter pylori,* significantly affecting microbial composition. Other acid‐resistant bacteria include *Streptococcus*, *Neisseria*, and *Lactobacillus.* Studies have identified dominant genera such as *Pasteurellaceae*, *Veillonella*, *Rothia*, *Streptococcus*, and *Prevotella* [22–24].
• Small intestine: The duodenum is colonized primarily by *Firmicutes* and *Actinobacteria,* while the jejunum hosts facultative anaerobes like *Lactobacillus*, *Enterococcus*, and *Streptococcus*. The ileum harbors a microbiota resembling that of the colon, consisting mainly of anaerobes and Gram‐negative organisms [21].
• Large intestine: Dominated by *Firmicutes* and *Bacteroidetes*, the colon also harbors opportunistic pathogens like *Bacteroides thetaiotaomicron* and *Bacteroides fragilis* [21].

### 3.2. Functions

The gut microbiota plays a crucial role in host health by contributing to various metabolic and immune processes.

• Dietary fiber metabolism: The gut microbiota ferments dietary fiber into SCFAs (e.g., acetate, propionate, butyrate), which regulate intestinal homeostasis and inflammation [1].
• Immunity: The gut microbiota plays a central role in shaping the immune system, promoting gut‐associated lymphoid tissue (GALT) development, and modulating systemic inflammation [19, 25].
• Lipid metabolism: Gut microbes influence energy balance, glucose metabolism, and lipid metabolism, impacting conditions like obesity and metabolic syndrome [26].
• Bile acid metabolism: Gut bacteria convert primary bile acids into secondary metabolites that regulate cholesterol homeostasis and lipid absorption [19].
• Tryptophan metabolism: The microbiota metabolizes tryptophan into bioactive compounds such as indoles, influencing immune function and inflammation [19].
• SCFAs: SCFAs modulate immune responses and maintain intestinal integrity by influencing regulatory T‐cell function and mucosal defense [19, 25–28].
• Choline metabolism: Gut bacteria convert choline into trimethylamine (TMA), which is oxidized into TMAO, a compound linked to CVD [1, 29].
• Gut–brain axis: The microbiota interacts with the enteric and central nervous systems, influencing neurodevelopment and cognitive function [30, 31].

### 3.3. Relationship Between Host and Microbes

The relationship between the host and gut microbiota is primarily commensal but can shift to pathogenic under dysbiotic conditions. The host employs feedback mechanisms to maintain microbial equilibrium, including immune modulation, spatial segregation of microbes, and alternative nutrient provision. Understanding the composition and function of the gut microbiota is essential for developing targeted interventions to improve CV and overall health [1].

## 4. Mechanism Linking Gut Microbiota and CV Health

Recent research underscores the significant role of gut microbiota in CV health, linking microbial composition alterations to various CVDs, such as myocardial infarction, hypertension, and atherosclerosis [32, 33]. The intricate relationship between gut microbiota and CV function, termed the “gut–heart axis,” [33] highlights the profound impact of gut health on CV outcomes. A deeper understanding of this bidirectional interaction is crucial for developing novel therapeutic strategies targeting gut microbiota to improve CV health.

### 4.1. Experimental Models of Causality

Fecal microbiota transplantation (FMT) serves as a critical experimental model to establish causality rather than a naturally occurring physiological mechanism. Germ‐free animal models have been instrumental in elucidating the functional role of gut microbiota in disease susceptibility. Studies have demonstrated significant increases in blood pressure following FMT from hypertensive individuals into germ‐free mice, thereby establishing a direct link between gut microbiota composition and hypertension [34, 35]. Additionally, specific bacterial species have been implicated in modulating CV health. For instance, the colonization of germ‐free ApoE‐deficient mice with the butyrate‐producing bacterium *Roseburia intestinalis* has been shown to reduce inflammatory markers and inhibit the progression of atherosclerotic lesions [36]. Furthermore, FMT between mouse strains with differing susceptibilities to atherosclerosis has revealed that gut microbiota composition plays a crucial role in disease development [37]. These findings emphasize the importance of both individual bacterial species and overall microbial community structure in influencing CVD risk.

### 4.2. Gut Microbiota Dysbiosis

Dysbiosis, an imbalance in microbial communities, lacks a universally standardized definition and can oversimplify complex microbial dynamics. For the purpose of this review, we define dysbiosis as a maladaptive imbalance in the microbial community structure, characterized by a loss of diversity, a reduction in beneficial commensals, and an expansion of pathobionts—that disrupts host–microbe homeostasis and contributes to disease pathology [38]. A healthy gut microbiota is characterized by a balanced ratio of *Bacteroidetes* to *Firmicutes*, which varies along the alimentary tract due to factors such as pH, oxygen levels, and nutrient availability [38]. Early studies utilizing 16S rRNA gene sequencing identified bacterial taxa associated with coronary artery disease (CAD) [39].

Research has shown that CAD patients exhibit elevated levels of *Lactobacillales* (*Firmicutes*) and reduced levels of *Bacteroidetes* (*Bacteroides* and *Prevotella*) [40]. Moreover, metagenomic analyses have found that individuals with atherosclerotic CVD harbor increased levels of *Enterobacteriaceae* and oral bacteria while displaying lower levels of butyrate‐producing bacteria [41]. HF patients demonstrate decreased microbial diversity, core microbiota depletion, and increased susceptibility to *Clostridium difficile* infections [42, 43]. Additionally, conditions of elevated right atrial pressure have been linked to increased mucosal biofilm formation, bacterial overgrowth, and colonization by pathogenic species such as *Candida*, *Campylobacter*, and *Shigella* [44]. Notably, reductions in butyrate‐producing bacteria, including *Faecalibacterium prausnitzii* and *Lachnospiraceae*, correlate with elevated inflammatory markers across HF patient populations [42, 45].

### 4.3. Immune Modulation

Growing evidence suggests that gut microbiota influence CV health by modulating immune cell activity, particularly T‐cell function [46, 47]. Microbiota transfer from inflammatory‐prone mice to atherosclerosis‐prone mice has been shown to accelerate atherosclerotic plaque formation, likely due to elevated systemic inflammatory cytokines such as interleukin (IL)‐1*β*, IL‐2, and interferon (IFN)‐*γ* [48, 49]. Conversely, supplementation with *Bacteroides vulgatus* and *Bacteroides dorei* has been found to reduce plasma tumor necrosis factor‐alpha (TNF‐*α*) levels and atherosclerotic lesions [49]. Similarly, administering *Lactobacillus plantarum* ATCC 14917 mitigated lesion formation by decreasing oxidized LDL, TNF‐*α*, and interleukin‐1 beta (IL‐1*β*) levels. The presence of *Roseburia* and *Blautia* has been associated with lower plasma cholesterol, TNF‐*α*, IL‐1*β*, and atherosclerotic burden [50, 51]. Additionally, dietary salt intake has been linked to hypertension via gut microbiota alterations. High salt consumption depletes *Lactobacillus murinus*, resulting in increased blood pressure, while supplementation restores balance by modulating T helper 17 (TH17) cell activity, thereby providing potential therapeutic avenues for hypertension management [52].

### 4.4. Barrier Dysfunction

The intestinal barrier, composed of mucus layers, tight junctions, and immune defenses, prevents bacterial translocation. This relationship is bidirectional: barrier compromise allows LPS translocation, but LPS itself, a structural component of Gram‐negative bacterial cell walls, can directly impair tight junction proteins, further increasing permeability and creating a vicious cycle of endotoxemia and inflammation [53]. Patients with CVD exhibit elevated endotoxin levels, with hepatic vein blood containing higher concentrations than ventricular blood [54, 55]. Certain bacterial species, such as *Akkermansia muciniphila*, help maintain intestinal integrity. Treatment with *A. muciniphila* has been shown to reduce endotoxemia and aortic atherosclerosis in ApoE‐deficient mice [56]. Human studies further support the therapeutic potential of pasteurized *A. muciniphila*, which was found to lower circulating LPS levels in obese individuals [57]. These findings suggest that strategies aimed at strengthening intestinal barrier function and modulating gut microbiota may offer promising avenues for CVD prevention and treatment.

### 4.5. Metabolite Dynamics

The gut microbiota functions as a metabolic organ, producing bioactive compounds that act as signaling molecules in the host’s systemic circulation. The balance between protective and pro‐atherogenic metabolites is a primary determinant of CV health.

SCFAs, produced primarily by the fermentation of dietary fibers by bacteria such as *Faecalibacterium* and *Roseburia* (including acetate, propionate, and butyrate) exert crucial cardioprotective effects. Mechanistically, SCFAs function as histone deacetylase (HDAC) inhibitors, which downregulate pro‐inflammatory gene expression in the vascular endothelium. Furthermore, they interact with G‐protein‐coupled receptors (GPR41 and GPR43) to regulate blood pressure via renin secretion control and vasodilation [58, 59]. Conversely, TMAOs represents a key pro‐atherogenic metabolite. Gut bacteria metabolize dietary nutrients containing TMA moieties, specifically choline, phosphatidylcholine, and L‐carnitine found in red meat and eggs into TMA [60]. TMA is absorbed and oxidized in the liver by flavin‐containing monooxygenases (FMO3) to form TMAO. TMAO promotes CV pathology through three distinct mechanisms: (1) inhibition of reverse cholesterol transport, (2) upregulation of scavenger receptors (CD36 and SR‐A1) on macrophages leading to foam cell formation, and (3) enhancement of platelet hyper‐reactivity and thrombosis risk [60–63].

**Figure 1:**
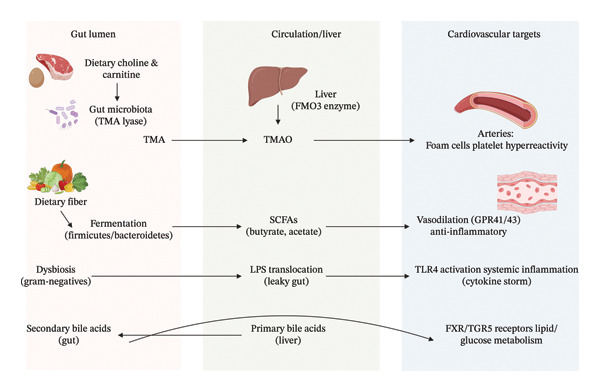
Integrated mechanistic pathways of the gut–heart axis.

Dysbiosis‐induced barrier dysfunction facilitates the translocation of LPS, an endotoxin, into the circulation. LPS binds to Toll‐like receptor 4 (TLR4) on endothelial and immune cells, triggering the NF‐κB inflammatory cascade and driving systemic inflammation [64, 65]. Additionally, gut bacteria modulate the bile acid pool; secondary bile acids act as signaling molecules on farnesoid X receptors (FXR) and TGR5, influencing lipid and glucose metabolism [65]. Collectively, these findings highlight the dual impact of gut microbiota metabolites on CV health, with SCFAs exerting protective effects while TMAO and LPS contribute to disease progression. Maintaining microbial balance is thus essential for CV health [60–65]. Figure 1 explores the integrated mechanistic pathway of the gut–heart axis, and Table 1 outlines a comprehensive summary of microbial influences on CV function.

**Table 1:** Key mechanisms of influence on CVD by gut microbiota.

Broad cause	Specific mechanisms	Summary of effects
Microbial Transplantation	Fecal transplants from CVD patients to germ‐free animals to establish causal links between microbiota composition and CVD phenotypes.	1. Demonstrates the direct role of microbiota in CVD and hypertension. 2. Beneficial effects were observed with specific anti‐inflammatory strains (e.g., *Roseburia intestinalis*).

Gut Microbiota Dysbiosis	Altered microbial diversity, depletion of beneficial taxa (e.g., *Roseburia* spp.), and overgrowth of pathogenic taxa (e.g., *Streptococcus* spp.).	1. Promotes systemic inflammation. 2. Reduces production of anti‐inflammatory metabolites (e.g., SCFAs). 3. Increases susceptibility to CVD.

Immune Modulation	Microbiota‐mediated changes in cytokine production (e.g., TNF‐*α*, IL‐1*β*) and T‐cell profiles.	1. Drives chronic inflammation in CVD. 2. Specific bacteria reduce inflammatory markers and protect against atherosclerosis.

Barrier Dysfunction	Loss of gut integrity allows translocation of endotoxins (e.g., LPS) into circulation, triggering inflammatory cascades.	1. Elevated endotoxemia was observed in CVD. 2. Contributes to systemic inflammation and atherogenesis.

Metabolite Dynamics	1. TMAO is derived from microbial metabolism of dietary choline and carnitine. 2. SCFAs from fiber fermentation. 3. Tryptophan metabolites affecting immune pathways.	1. TMAO is linked to vascular dysfunction and atherosclerosis. 2. SCFAs lower blood pressure via receptor modulation. 3. Tryptophan metabolites regulate inflammatory responses.

Abbreviations: CVD, Cardiovascular Disease; IL‐1*β*, Interleukin 1 beta; LPS, Lipopolysaccharide; SCFAs, Short‐Chain Fatty Acids; TMAO, Trimethylamine N‐oxide; TNF‐α, Tumor Necrosis Factor alpha.

## 5. Gut Microbiota and Specific CVDs

### 5.1. Atherosclerosis

Atherosclerosis is a chronic inflammatory condition driven by dysregulated lipid metabolism and an inappropriate immune response, leading to cholesterol‐rich macrophage accumulation within arterial walls [66]. Research utilizing 16S rRNA gene pyrosequencing has revealed that atherosclerotic plaques contain bacterial DNA with phylotypes commonly found in the gut microbiota. The quantity of bacterial DNA in plaques is correlated with inflammation, suggesting potential microbial translocation [66, 67]. Certain bacteria, such as *Streptococcus* and *Veillonella*, can directly influence plaque composition, while *Pseudomonas*, *Klebsiella*, and *Chlamydia pneumoniae* have also been identified within atherosclerotic lesions [68]. However, most studies have not established a definitive link between plaque microbiota composition and clinical outcomes such as plaque rupture or CV events [68–70].

Metabolically, the progression of atherosclerosis is heavily influenced by the TMAO pathway described in Section 4.5. Elevated plasma TMAO levels are strongly correlated with increased atherosclerotic burden and major adverse cardiovascular events (MACE) in humans [71]. In animal models, dietary suppression of TMA generation attenuates plaque formation, confirming a causal role [72]. Conversely, the depletion of SCFA‐producing bacteria reduces the availability of butyrate, removing its protective inhibition of vascular inflammation and accelerating plaque progression [72–74].

### 5.2. Hypertension

Hypertension is a major modifiable risk factor for CVD and is strongly associated with gut microbiota dysregulation [75, 76]. Alterations in microbial composition include an increased *Firmicutes*‐to‐*Bacteroidetes* (F/B) ratio, a reduction in SCFA‐producing bacteria, an expansion of lactic acid bacteria, and an increase in *Proteobacteria* and *Cyanobacteria* [69].

Mechanistically, the reduction of SCFAs impairs blood pressure regulation. As noted in Section 4.5, SCFAs normally interact with GPR41 to mediate vasodilation. In hypertensive animal models, reduced SCFA availability leads to increased peripheral vascular resistance and vascular remodeling [77–80]. Furthermore, high salt intake, a known risk factor for hypertension, has been shown to deplete Lactobacillus murinus; supplementing with this bacterium prevents salt‐sensitive hypertension by modulating T helper 17 (TH17) cell activity. Conversely, elevated TMAO levels contribute to hypertension by prolonging the pressor effects of angiotensin II and inducing endothelial dysfunction via oxidative stress [81–83]. Probiotic interventions aimed at restoring the SCFA‐producing population have shown promise in lowering systolic blood pressure by mitigating these vascular inflammatory responses [84].

### 5.3. Arrhythmia

**Figure 2:**
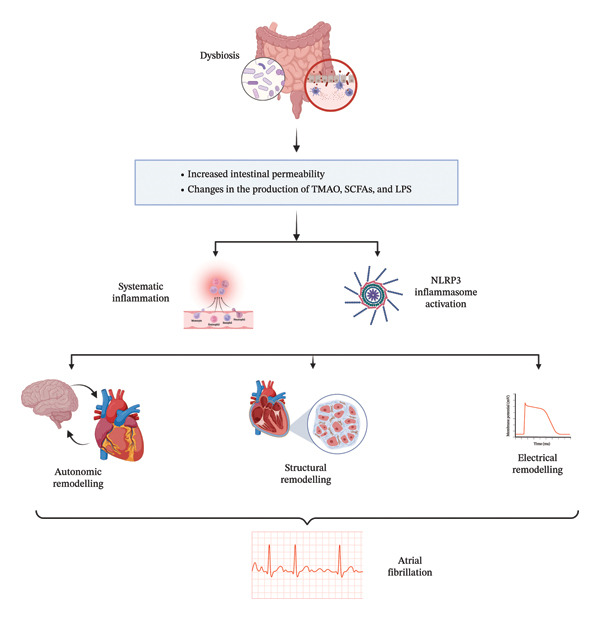
Relationship between gut microbiota, inflammation, and atrial fibrillation.

Atrial fibrillation (AF) is the most common sustained arrhythmia and is increasingly linked to the systemic inflammation driven by gut dysbiosis. The “gut‐heart” connection in AF is mediated primarily through inflammatory signaling and structural remodeling of the atria [85]. Dysbiosis contributes to AF progression by compromising the intestinal barrier, leading to elevated circulating LPS [85–87]. LPS activates NLRP3 inflammasomes in atrial cardiomyocytes, promoting the release of pro‐inflammatory cytokines IL‐1*β* and IL‐18. This inflammatory milieu promotes atrial fibrosis, a substrate for re‐entrant arrhythmias [86]. Additionally, elevated TMAO levels have been implicated in enhancing atrial autonomic remodeling and increasing susceptibility to AF [88–90]. TMAO promotes M1 macrophage infiltration in atrial tissue, exacerbating inflammation and fibrosis. Conversely, bile acids modulate this risk; the activation of FXR by secondary bile acids has been shown to inhibit the inflammatory cascades that trigger AF, suggesting that maintaining a healthy bile acid metabolism is protective against arrhythmia development [91–94]. Figure 2 illustrates the relationship between gut microbiota, inflammation, and AF.

### 5.4. HF

HF is primarily caused by myocardial dysfunction resulting from conditions such as CAD, hypertension, and metabolic disorders [95]. Emerging evidence highlights a bidirectional relationship between gut microbiota and HF. Dysbiosis induces systemic inflammation, oxidative stress, and metabolic dysregulation, exacerbating myocardial dysfunction [95, 96].

According to the “gut hypothesis of HF,” alterations in gut permeability and microbiota composition—caused by reduced cardiac output, intestinal edema, and altered gut motility—worsen HF outcomes [97]. Patients with HF exhibit increased intestinal colonization by pathogenic bacteria such as *Campylobacter*, *Shigella*, and *Salmonella*, leading to enhanced systemic inflammation [44]. Additionally, LPS binds to Toll‐like receptor 4 (TLR4) on cardiomyocytes, cardiac fibroblasts, and macrophages, promoting pro‐inflammatory cytokine release and myocardial damage via NLRP3 inflammasome activation [98, 99].

SCFA‐producing bacteria, including *Eubacterium*, *Faecalibacterium*, and *Ruminococcus*, are reduced in HF patients, whereas overgrowth of proinflammatory *Proteobacteria* is commonly observed [100, 101]. Restoring gut microbiota balance through probiotic or prebiotic interventions has demonstrated improvements in inflammatory markers and clinical outcomes [102]. TMAO contributes to myocardial hypertrophy and fibrosis by activating Smad3 signaling, leading to left ventricular dilation and increased brain natriuretic peptide levels. Additionally, inflammaging, a chronic low‐grade inflammatory state associated with aging, has been linked to gut dysbiosis in elderly HF patients [103–105].

### 5.5. Stroke

Gut microbiota and its metabolites regulate the progression of neurological disorders, including ischemic stroke [106]. Dysbiosis alters the intestinal environment, affecting nutrient absorption, immune homeostasis, and systemic metabolism, thereby increasing the risk of ischemic stroke via mechanisms such as hypertension and diabetes [107]. Furthermore, stroke severity and prognosis are influenced by gut microbiota alterations, which modulate immune responses, inflammation, and thrombosis [108].

The microbiota–gut–brain axis facilitates bidirectional communication between the gut and central nervous system through neural, endocrine, and immune pathways [109–111]. Microbial metabolites such as SCFAs contribute to brain–gut barrier integrity, mitigate oxidative stress, and reduce neuroinflammation following an acute ischemic stroke (AIS) [112]. Additionally, poststroke bacterial translocation may contribute to stroke‐associated infections, with autonomic nervous system overactivation and excessive glucocorticoid release exacerbating gut dysbiosis and intestinal permeability [113].

### 5.6. Pulmonary Thromboembolism

Dysbiosis has been implicated in increased venous thromboembolism risk, particularly by reducing beneficial commensal bacteria and an overgrowth of pathogenic Gram‐negative bacteria, such as *Enterobacteriaceae*. LPS, a glycolipid component of the Gram‐negative bacterial outer membrane, induces hypercoagulability by binding Toll‐like receptors on endothelial cells and platelets, activating the coagulation cascade. TMAO enhances platelet hyper‐reactivity and promotes thrombus formation, further increasing pulmonary thromboembolism susceptibility [114].

Recent research has explored prebiotic and probiotic interventions to counteract gut dysbiosis‐related thrombogenesis, with resveratrol emerging as a promising candidate for mitigating hypercoagulability and reducing thrombotic risk [115].

## 6. Influence of Diet and Lifestyle on Gut Microbiota and CV Health

The composition and function of gut microbiota are profoundly influenced by diet, but other lifestyle factors, including exercise and sleep, also play a crucial role. The dynamic interplay between dietary habits, gut health, and immune function has been well documented, with growing evidence supporting the impact of gut microbiota on CV health [116]. Diet‐driven changes in gut microbial populations can influence susceptibility to infections and systemic inflammation, thereby affecting CV outcomes.

The human GI tract hosts a diverse microbial community, predominantly residing in the large intestine, where the fermentation of undigested carbohydrates, proteins, and fibers produces SCFAs [117]. These SCFAs, such as butyrate, acetate, and propionate, are essential in modulating immune responses and metabolic pathways. The predominant bacterial genera in the large intestine include *Bifidobacterium, Lactobacillus, Bacteroides, Clostridium, Escherichia, Streptococcus*, and *Ruminococcus*. Given the intricate relationship between gut microbiota and CV homeostasis, lifestyle choices that alter gut microbial composition can significantly impact the gut–heart axis [116–118].

### 6.1. Mediterranean Diet (MD)

MD has been extensively studied for its CV benefits, primarily due to its emphasis on plant‐based foods such as legumes, whole grains, nuts, fruits, vegetables, and extra virgin olive oil. This diet is rich in omega‐3 polyunsaturated fatty acids (PUFAs), which exert antiatherogenic and anti‐inflammatory effects by activating peroxisome proliferator‐activated receptor gamma (PPAR‐γ) and inhibiting nuclear factor kappa B (NF‐κB), thereby reducing the production of pro‐inflammatory cytokines like TNF‐*α*. As a result, adherence to the MD is associated with a reduced risk of CAD, inflammatory bowel disease, and other chronic inflammatory conditions [119, 120].

Beyond its direct CV benefits, the MD promotes gut microbial diversity. Its high fiber content fosters the growth of *Bacteroides* species and enhances gut acidification, which supports a balanced microbial ecosystem [120, 121]. Moreover, the fermentation of dietary fibers in the MD produces SCFAs, particularly butyrate, which has been linked to lower rates of CVD and even some cancers [122]. A controlled trial by Kimble et al. demonstrated that MD consumption increases the abundance of *Adlercreutzia equolifaciens*, a bacterium involved in polyphenol metabolism, and *F. prausnitzii*, a butyrate‐producing species with potent anti‐inflammatory properties [121–123].

### 6.2. Western Diet (WD)

In contrast, WD—characterized by high intakes of saturated fats, refined carbohydrates, sugars, processed foods, and red meats—has been associated with dysbiosis and adverse CV outcomes [121, 122]. Prolonged consumption of WD promotes the secretion of LPS and chylomicron accumulation, fostering an increased abundance of Gram‐negative bacteria such as *Alistipes* and *Bacteroides*. This shift compromises intestinal tight junction integrity, facilitating LPS translocation into the bloodstream and triggering systemic inflammation through elevated TNF‐*α*, interferon‐gamma (IFN‐*γ*), and IL‐1*β*. Over time, this chronic inflammatory state contributes to endothelial dysfunction, hypertension, and atherosclerosis [120, 124, 125].

A 3‐year study by Tang et al. found that high dietary phosphatidylcholine—a component abundant in WD—leads to the production of TMAO, a gut‐derived metabolite strongly associated with an increased risk of atherosclerosis [126]. Additionally, the combination of a sedentary lifestyle and excessive consumption of sugar‐sweetened beverages and alcohol further exacerbates gut barrier disruption. A study by Dr. Leclercq on 60 patients demonstrated that alcohol consumption significantly alters gut microbial balance, leading to long‐term cardiac and metabolic dysfunction [127].

### 6.3. Polyphenols and Gut Health

Dietary polyphenols, found in plant‐based foods such as fruits, vegetables, tea, cocoa, and wine, have been widely studied for their antioxidant and cardioprotective properties [128, 129]. These compounds enhance gut microbial diversity by promoting the growth of beneficial bacteria like *Bifidobacterium* and *Lactobacillus*. Red wine polyphenols, in particular, have been linked to increased levels of *Bacteroides*, a genus associated with metabolic health benefits [128, 129].

Polyphenol‐rich diets act as prebiotics, fostering the activity of beneficial microbes and improving nutrient absorption [128–130]. *Bifidobacterium*, a common probiotic, has been shown to support immune function, protect against inflammatory bowel disease, and reduce cancer risk. Additionally, cocoa‐derived polyphenols have been associated with increased high‐density lipoprotein (HDL) levels and reduced plasma triacylglycerol and inflammatory markers [129, 130]. Furthermore, polyphenols exert antibacterial activity against harmful pathogens like *Staphylococcus aureus* and *Salmonella* while limiting the growth of pathogenic *Clostridium* species [130]. Hydroxycinnamic acid, a polyphenol subtype, has demonstrated hepatoprotective, antiobesity, and CV benefits [129–131]. Moreover, flavonoids from polyphenol‐rich foods act as free radical scavengers, reducing blood lipid levels and cholesterol [132].

### 6.4. Exercise and Microbiota Modulation

Physical activity is another crucial modulator of gut microbiota composition. Regular exercise enhances microbial diversity and improves the *Bacteroidetes*‐to‐*Firmicutes* ratio, which is linked to better weight management and metabolic health. Exercise promotes the proliferation of beneficial microbes that reinforce gut barrier integrity and mucosal immunity [133].

During exercise, skeletal muscle contractions stimulate the release of myokines, such as interleukin‐6 (IL‐6), which subsequently enhance the secretion of glucagon‐like peptide‐1 (GLP‐1), a key regulator of glucose metabolism [133, 134]. Exercise also increases SCFA production, leading to improved gut integrity, reduced systemic inflammation, and enhanced metabolic function [134, 135]. A randomized controlled trial (RCT) by Zhong et al. demonstrated that aerobic exercise in women significantly improved gut microbiota diversity, with beneficial microbial shifts associated with reduced CV risk factors. Using 16S rRNA sequencing, the study identified increased health‐promoting bacterial genera, reinforcing the connection between physical activity, gut health, and CV well‐being [136].

## 7. Therapeutic Interventions

The gut microbiota, a complex ecosystem of microorganisms residing in the GI tract, plays a pivotal role in overall health, including CV well‐being. This has spurred growing interest in therapeutic interventions targeting the gut microbiota as a means to prevent and manage CVDs. Current strategies include probiotics, prebiotics, FMT, and pharmacological approaches.

### 7.1. Probiotics and Prebiotics

Probiotics—live microorganisms that confer health benefits—and prebiotics—nondigestible food components that promote the growth of beneficial bacteria—have been extensively studied for their cardioprotective effects. Clinical trials demonstrate their efficacy in modulating CV risk factors. For instance, a trial in patients with type 2 diabetes mellitus (T2DM) found that a 6‐week course of probiotic supplementation significantly reduced blood pressure and the Framingham risk score [137]. Similarly, a meta‐analysis confirmed significant reductions in blood pressure, total cholesterol, LDL‐C, serum glucose, HbA1c, and BMI alongside increased HDL‐C levels [138]. These effects were particularly pronounced with prolonged interventions, higher dosages, and in individuals with metabolic disorders.

The benefits of probiotics are strain‐specific. Strains such as *Lactobacillus acidophilus, L. plantarum, L. fermentum*, and *L. gasseri* have demonstrated improvements in blood pressure and lipid profiles. Additionally, *L. rhamnosus* GR‐1 and *Limosilactobacillus reuteri* RC‐14 contribute to urogenital health in women, indirectly supporting overall well‐being [139]. Multistrain probiotic formulations often exhibit superior efficacy compared to single‐strain interventions, enhancing CV health outcomes more effectively. Specific strains like *Limosilactobacillus reuteri* TF‐7, *Enterococcus faecium* TF‐18, and *Bifidobacterium animalis* TA‐1 have demonstrated hypocholesterolemic effects, synergistically improving lipid profiles and gut microbiota composition [140, 141]. The mechanisms underlying these benefits include SCFA production, bile salt hydrolase activity, lipid metabolism regulation, and modulation of systemic inflammation, all of which contribute to improved CV health [141].

### 7.2. FMT

FMT, the process of transferring gut microbiota from a healthy donor to a recipient, is an emerging intervention primarily used for *Clostridioides difficile* infections. Its potential in CV health is under investigation. Preclinical studies suggest that FMT can influence atherosclerosis development by modifying gut microbiota composition and reducing systemic inflammation [111]. However, human studies remain limited.

Potential CV benefits of FMT include improved metabolic profiles, modulation of gut‐derived inflammation, and reduction in inflammatory markers associated with metabolic syndrome. Despite its promise, FMT presents several challenges, including limited direct evidence for CV disease, risks of infection transmission, variability in outcomes, and ethical and regulatory concerns. Further research with standardized protocols and large‐scale clinical trials is needed to establish its efficacy and safety for CV applications [142].

### 7.3. Pharmacological Approaches

Targeting gut microbial metabolites offers another avenue for CV intervention. One of the most well‐researched microbial metabolites is TMAO, which is derived from dietary choline and carnitine metabolism by gut bacteria. Elevated TMAO levels have been linked to atherosclerosis and adverse CV events [143]. Pharmacological strategies to reduce TMAO include [144]

1. inhibiting TMA production by targeting microbial enzymes responsible for its generation,
2. blocking the hepatic conversion of TMA to TMAO, and
3. using nonlethal inhibitors that selectively suppress TMA‐producing pathways without disrupting overall gut microbiota balance.

Reducing TMAO levels has shown promise in lowering CV risk factors, including platelet reactivity and thrombosis risk. Other gut‐derived metabolites, such as SCFAs and bile acids, also play crucial roles in CV health, offering additional therapeutic opportunities through diet, probiotics, and pharmacological agents [144–146].

### 7.4. Emerging Therapeutic Strategies

The gut–heart axis underscores the role of gut microbiota in regulating host metabolism, inflammation, and immune responses. Emerging therapeutic strategies target bacterial metabolites, enhance intestinal barrier function, and modulate inflammatory pathways [147].

Next‐generation probiotics (NGPs), engineered for enhanced stability and targeted disease management, represent a promising advancement in microbiota‐based therapy. The integration of synthetic biology and bioinformatics is facilitating more precise interventions [148, 149]. Future directions include [147–149]

• personalized microbiota‐based therapies tailored to individual gut microbiome profiles,
• multistrain probiotic formulations optimized for CV benefits,
• advanced probiotic delivery systems ensuring greater efficacy, and
• combined prebiotic and probiotic interventions to maximize gut microbiota modulation.

## 8. Challenges

Despite the growing recognition of gut microbiota’s role in CV health, several key challenges remain. One of the foremost difficulties is distinguishing causation from correlation. While numerous studies have established associations between gut dysbiosis and CVD, definitive proof that gut microbiota alterations directly contribute to the development or progression of CVD is still lacking. Large‐scale, longitudinal studies and interventional RCTs targeting microbiota modulation are needed to clarify causal relationships. To highlight the translational gap, Table 2 contrasts the causal mechanisms established in murine models with the clinical associations observed in human studies.

**Table 2:** Contrast of key findings in animal models vs. human clinical studies.

Disease domain	Animal model findings (mechanistic causality)	Human study findings (clinical association/intervention)
Hypertension	1. FMT from hypertensive humans into germ‐free mice significantly increases blood pressure in the mice. 2. Mechanism: Salt‐sensitive hypertension is linked to *Lactobacillus* murinus depletion; supplementation prevents BP rise via TH17 modulation.	1. Association & Intervention: Probiotic supplementation (e.g., *L. acidophilus*, *L. plantarum*) in patients with T2DM significantly reduced systolic blood pressure. 2. Observation: High salt intake correlates with altered gut microbiota, but direct mechanistic pathways are harder to isolate than in murine models.

Atherosclerosis	1. Germ‐free ApoE‐deficient mice colonized with *Roseburia* intestinalis showed reduced atherosclerotic lesions. 2. Dietary suppression of TMA generation in mice directly attenuates plaque formation.	1. Elevated plasma TMAO levels are strongly correlated with MACE and atherosclerotic burden. 2. Microbial Signatures: CAD patients harbor increased *Enterobacteriaceae* and *Streptococcus* species.

Metabolic/TMAO	High dietary choline/phosphatidylcholine in mice drives TMAO production and subsequent vascular inflammation.	Controlled feeding studies confirm that high‐fat/Western diets increase TMAO production in humans. Reduction of TMAO is associated with lower thrombosis risk.

Inflammation/barrier function	Endotoxemia: Akkermansia muciniphila treatment in ApoE‐deficient mice reduces endotoxemia (LPS) and aortic atherosclerosis.	1. Pasteurized A. muciniphila lowers circulating LPS and improves metabolic parameters in obese humans. 2. Heart failure patients show higher hepatic vein endotoxin levels compared to ventricular blood, indicating gut translocation.

Abbreviations: ApoE, Apolipoprotein E; BP, Blood Pressure; CAD, Coronary Artery Disease; FMT, Fecal Microbiota Transplantation; LPS, Lipopolysaccharide; MACE, Major Adverse Cardiovascular Events; T2DM, Type 2 Diabetes Mellitus; TH17, T helper 17 cells; TMA, Trimethylamine; TMAO, Trimethylamine N‐oxide.

Another significant hurdle is elucidating the precise mechanisms underlying the gut–heart axis. Proposed pathways such as TMAO production, SCFAs, and inflammation play integral roles, but their interactions and relative contributions remain unclear. Multiomics approaches integrating genomics, metabolomics, and proteomics are essential to unravel the complex interplay between gut microbiota, host metabolism, and CV function. Identifying key microbial species and their metabolites will be critical in refining therapeutic targets.

Interindividual variability in gut microbiota composition further complicates the development of universal interventions. Factors such as genetics, diet, lifestyle, and environmental exposures contribute to significant heterogeneity, necessitating a more personalized approach. Stratifying individuals based on microbiota profiles may enhance intervention efficacy, but this requires advanced profiling techniques and robust data analysis methods. Additionally, inconsistencies in study designs, sample collection, sequencing technologies, and data interpretation hinder reproducibility. Establishing standardized protocols for gut microbiota research, including uniform methodologies for assessing gut barrier function and systemic inflammation, is crucial for generating reliable and comparable data.

Translating microbiota research into effective clinical therapies also presents considerable challenges. Identifying optimal probiotic strains, prebiotics, and microbiota‐modulating strategies, along with developing efficient delivery systems, remains an ongoing endeavor. Long‐term safety and efficacy must be established through extensive RCTs, with careful consideration of potential adverse effects. Furthermore, the intricate interplay between diet, lifestyle, and gut microbiota makes it challenging to isolate the specific contributions of microbiota modifications to CV outcomes.

**Figure 3:**
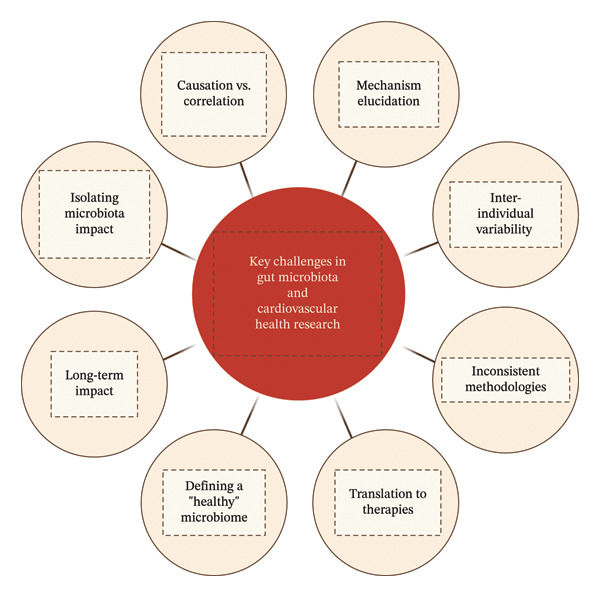
Key challenges in gut microbiota and cardiovascular health research.

Finally, the long‐term impact of gut microbiota modulation on CV health is largely unknown. While short‐term studies have demonstrated promising results, longitudinal research is needed to assess the durability of interventions and their influence on CVD onset, progression, and prognosis. Addressing these challenges will be pivotal in unlocking the full potential of gut microbiota–based strategies for CVD prevention and treatment. Figure 3 summarizes the key challenges.

## 9. Future Directions

Advancing our understanding of the gut microbiota’s role in CV health requires a multifaceted approach, integrating emerging technologies and novel therapeutic strategies. Precision medicine will play a central role by incorporating gut microbiota profiling alongside clinical and multiomics data, enabling tailored interventions for CVD prevention and management.

Developing NGPs with enhanced functionality and targeted delivery mechanisms holds promise for improving therapeutic efficacy. Engineered probiotics capable of producing specific metabolites or colonizing targeted gut sites may offer more precise interventions. Similarly, FMT protocols need optimization for CVD applications, focusing on identifying beneficial microbial consortia, ensuring safety, and standardizing donor selection and stool processing.

Pharmacological strategies targeting harmful microbial metabolites or modulating gut microbiota composition present another exciting avenue. Identifying novel drug targets and selective inhibitors could mitigate adverse CV effects associated with dysbiosis. A systems biology approach, integrating multiomics data with computational modeling, will be crucial in deciphering the complex interactions between gut microbiota, host metabolism, and the CV system, ultimately facilitating the identification of key therapeutic pathways.

To translate these advancements into clinical practice, large‐scale RCTs are essential to assess the safety and efficacy of gut microbiota‐modulating interventions. Further research into the gut–heart axis will be critical in uncovering underlying mechanisms and novel therapeutic targets, particularly in relation to blood pressure regulation, inflammation, and other CV processes.

Finally, a preventative approach focusing on early‐life interventions to promote a healthy gut microbiota may have profound implications for long‐term CV health. Investigating the microbiota’s role in CV development and identifying strategies to optimize gut health from a young age could be a pivotal step in reducing CVD risk later in life.

## 10. Conclusion

The gut microbiota is no longer viewed merely as a commensal ecosystem but as a modifiable determinant of CV pathology. The “gut–heart axis” operates through defined molecular pathways, principally the balance between protective SCFAs and pro‐atherogenic mediators such as TMAO and LPS. These microbial signals are integral to the pathogenesis of atherosclerosis, hypertension, and arrhythmias, representing a significant therapeutic frontier.

However, translating these mechanistic insights into clinical utility requires overcoming substantial limitations. Current paradigms rely heavily on murine models that do not fully recapitulate human CV physiology or microbiome complexity. Future research must prioritize (1) the standardization of “dysbiosis” definitions to ensure reproducibility; (2) a shift from cross‐sectional association studies to longitudinal, interventional human trials capable of establishing causality; and (3) the application of multiomics to account for interindividual variability.

Ultimately, the evolution of CV medicine will likely necessitate treating the microbial ecosystem as a functional organ. By integrating microbiome profiling into risk stratification, we may advance toward precision therapies, ranging from NGPs to selective metabolite inhibitors that target the gut–heart axis to improve long‐term CV outcomes. Looking ahead, advances in precision medicine, NGPs, and systems biology approaches hold the potential to refine our understanding of the gut–heart axis. By integrating these insights into clinical practice, researchers and clinicians can develop more effective, personalized interventions that improve CV outcomes and overall health. As the field continues to evolve, unlocking the full therapeutic potential of gut microbiota may pave the way for innovative strategies to combat CVD and enhance long‐term well‐being.

## Author Contributions

Conceptualization, Maneeth Mylavarapu, Israel Garcia, and Harshaman Kaur; literature review, Maneeth Mylavarapu, Harendra Kumar, and Kiyan Ghani Khan; writing–original draft preparation, Maneeth Mylavarapu, Harshaman Kaur, Roopeessh Vempati, Harendra Kumar, Sai Lakhan Kyasa, Kiyan Ghani Khan, Sriharsha Dadana, Israel Garcia, Fabio Enrique Parada Cabrera, Amninder Singh, Sai Lakhan Kyasa, and Vikramjit S. Purewal; writing–review and editing, Maneeth Mylavarapu, Sriharsha Dadana, Fabio Enrique Parada Cabrera, and Amninder Singh; visualization, Maneeth Mylavarapu; supervision, Maneeth Mylavarapu; project administration, Maneeth Mylavarapu, Israel Garcia, Amninder Singh, and Harshaman Kaur; review and editing (post peer‐review), Maneeth Mylavarapu, Israel Garcia.

## Funding

This research received no external funding.

## Disclosure

All authors have read and agreed to the revised version of the manuscript.

## Conflicts of Interest

The authors declare no conflicts of interest.

## Data Availability

Data sharing is not applicable to this article as no new data were created or analyzed in this study.
